# High-Resolution Leaf Image Sequences with Geometric Alignment for Dynamic Phenotyping of Foliar Diseases

**DOI:** 10.1038/s41597-026-06567-y

**Published:** 2026-01-23

**Authors:** Jonas Anderegg, Bruce A. McDonald

**Affiliations:** Plant Pathology Group, Institute of Integrative Biology, ETH Zurich, Zurich, Switzerland

**Keywords:** Biotic, Image processing

## Abstract

Time-resolved phenotyping of disease symptoms enables dissection of resistance mechanisms and improves diagnosis, but acquiring phenotypic data at satisfactory scale remains challenging. Advances in imaging and image processing have improved measurement precision, robustness, and throughput, but further improvements are needed for practical application. We present a data set comprising 12,520 high-resolution (~0.03 mm/pixel) RGB images representing 1,032 time series of wheat leaves with developing disease symptoms. All images are geometrically aligned with a median precision of 0.16 mm (≈5 pixels). The dataset includes transformation matrices, symptom segmentation masks, metadata on treatments, weather, crop phenology, and disease occurrence, and a lightweight Python toolkit for loading, aligning, inspecting, and editing image sequences. These resources enable detailed investigation of leaf-level disease dynamics such as lesion, pustule, and fruiting body emergence rates, lesion growth, and dynamic interactions of disease development with spatial and environmental contexts. They offer a broad basis for developing improved methods for image alignment and symptom detection, segmentation, and tracking, possibly by tackling these connected challenges within a single end-to-end framework.

## Background & Summary

Monitoring plant health over time in realistic field environments enables tracking of disease progression and investigating its dependence on factors like host genotype, environmental conditions, and pathogen biology. Disease progression is commonly assessed at the canopy scale, either through repeated visual inspection or, more recently, using remote or proximal sensing techniques, which typically provides an integrated measure of disease severity per experimental unit^1–5^. In many pathosystems, such integrated assessments at the canopy level reflect a cumulative outcome of multiple underlying epidemic processes that include the establishment of new infections, emergence and expansion of lesions, and the production and spread of secondary inoculum over time^6^. While such integrated assessments are essential for resistance breeding and disease management in commercial production, they offer limited insights into the epidemic processes driving disease development and the degree to which they are affected by host-, environment-, and pathogen-related factors^7–11^. As a result, important questions remain unresolved: How do individual components of resistance such as a reduced infection frequency, an extended latent period, slowed lesion expansion, or reduced spore production contribute to epidemic development under fluctuating environmental conditions? To what extent do these components vary among host genotypes? Could variation be leveraged in breeding for disease resistance, and what are the underlying genetic, molecular, or physiological mechanisms? While finding answers to these questions presents multiple challenges, a major initial obstacle is the lack of precise and high-throughput phenotyping techniques to measure and dissect these components under field conditions^7,12^. Previous approaches for monitoring stress responses at the level of individual plant organs typically relied on time-consuming and potentially subjective manual assessments^12^, or used fixed installations of cameras capturing image sequences of fixed leaves^13–15^. These approaches ensure that plant organs are properly aligned with the focal plane and field-of-view of the camera, simplifying the processing and analysis of image sequences. However, both approaches offer very limited scalability which makes their use in large experiments impractical. To increase measurement throughput, improved methods are required that can effectively handle image data with a lower level of standardization that can be collected with less effort^16^.

**Fig. 1:**
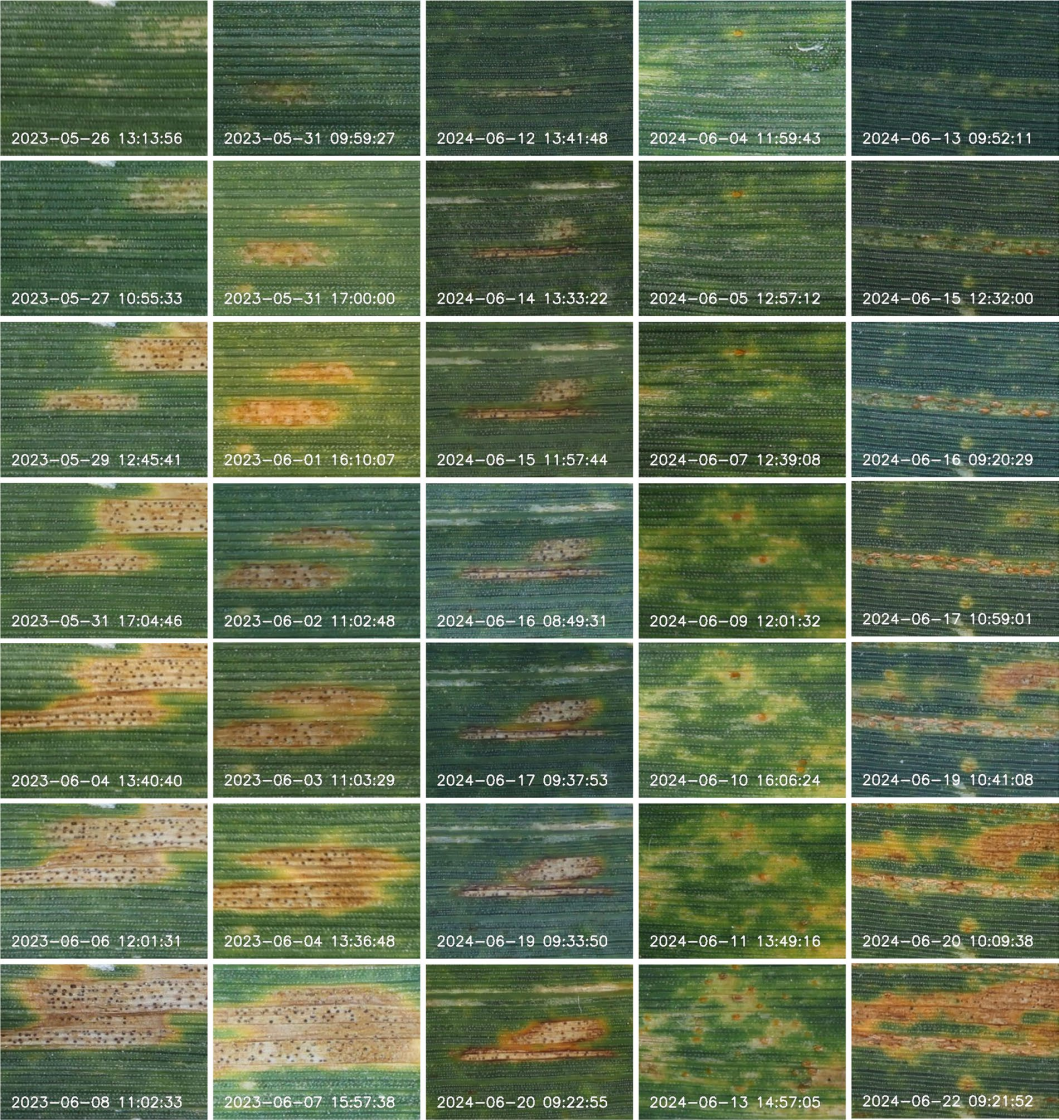
Example cut-outs from geometrically aligned leaf image sequences. Time points of image acquisition are labeled in white. Only a subset of all available images in the sequences is shown. The first three columns show developing lesions caused by *Zymoseptoria tritici* with dark fruiting bodies (pycnidia); the fourth column shows developing brown rust symptoms caused by *Puccinia triticina*, recognizable by the seemingly random distribution of individual pustules; the fifth column shows developing strip rust symptoms caused by *Puccinia striiformis* f.sp. *tritici* recognizable as linearly aligned pustules. The later images in columns 4 and 5 display mixed infections with *Z. tritici*.

**Fig. 2:**
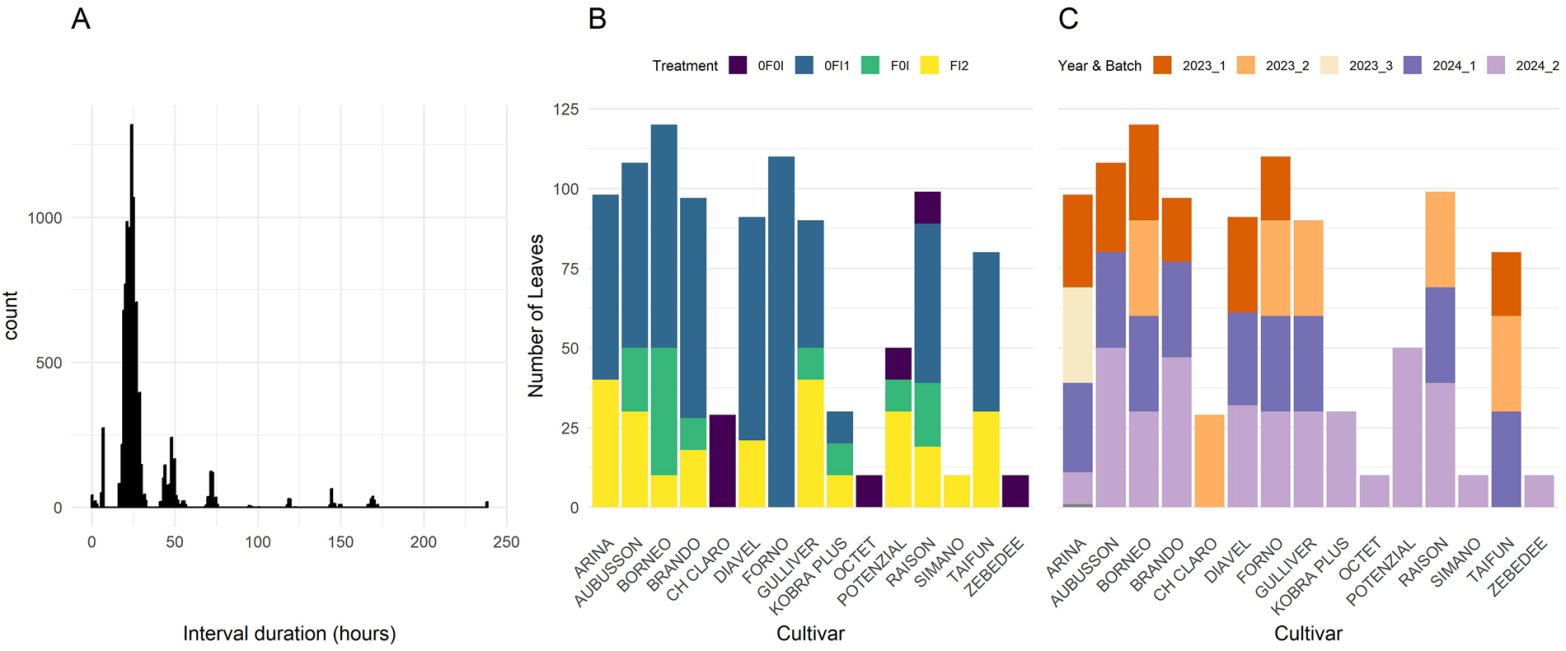
Overview of the dataset structure. (**A**) Distribution of time lags between consecutive imaging of the same leaves. Most intervals are approximately 24 hours. (**B**) Sample distribution by cultivar and treatment for pooled data from both years. (**C**) Sample distribution by year and measurement batch, for pooled data from all treatments. Batch 1 corresponds to penultimate leaves that were monitored first; Batch 2 and Batch 3 correspond to flag leaves that were monitored later. Modified from Anderegg *et al*.^8^.

In the context of this challenge, we present a dataset of 12,520 manually captured RGB images of fully grown wheat leaves. These represent 1,032 leaf image sequences with near-daily resolution obtained directly in the field. Most image sequences capture the development of foliar diseases from the appearance of first symptoms to high disease severity levels, particularly for brown rust (caused by *Puccinia triticina*), yellow rust (caused by *Puccinia striiformis* f.sp. *tritici*), and Septoria tritici blotch (caused by *Zymoseptoria tritici*; Fig. 1). All images share a constant spatial resolution of ~0.03 mm/pixel. On average, each sequence contains 12.1 ± 3.4 images taken at 30.6 ± 25.1 hour intervals, spanning 14.9 ± 3.2 days (Fig. 2A). The dataset includes leaves from 15 wheat cultivars (Table 1) subjected to various fungicide and inoculation treatments, imaged in 2-3 consecutive batches that correspond to different time spans in the growing season. The distribution of the samples according to cultivars, treatments, and measurement batches is shown in Fig. 2B,C. All images are registered to the first image in each sequence using piecewise affine transformations guided by artificial markers that were manually added to the leaf surface. The dataset used to train the reference mark detection model is also made available to support eventual re-training. All images were processed with key point detection and semantic segmentation models to identify disease symptoms. Instance masks tracking individual lesions through each image sequence are available.

**Table 1:** List of the wheat cultivars included in the field experiment.

GenName	FH	GS55	Onsen	Awns	Fl0Ang	Fl0Glc	PLACL	TraitComb	Criterion	Originator	Accession Number	Recommended for
GULLIVER	0.76	221	641	0	1	0	20.3	LL	TraitComb	Nickerson International Research GEIE	na	GBR
KOBRA PLUS	0.84	219	593	0	1	3	48.9	LL	TraitComb	Hodowla Roslin Rolniczych Nasiona Kobierzyc Sp.z o. o.	RICP-0C0107039	POL
TAIFUN	0.85	216	656	0	5	3	37.8	HL	TraitComb	Lochov-Petkus GMBH	K-57182; AUS-22191	DEU, HUN, LTU, DNK
RAISON	0.75	222	579	0	1	8	29.6	LH	TraitComb	na	na	FRA
BRANDO	0.71	222	586	0	1	8	41.8	LH	TraitComb	Cambridge PB Twyford	K-64522	GBR
POTENZIAL	0.82	221	699	0	2	9	22.1	LH	TraitComb	Deutsche Saatveredelung AG, OT Leutewitz	RICP-01C0107156	DEU, CZE, DNK
DIAVEL	0.86	218	600	0	5	8	na	HH	TraitComb	Agroscope/DSP (Federal Research Station for Agronomy)	na	CHE
BORNEO	0.74	221	523	0	6	7	42.8	HH	TraitComb	Saatzucht J.Breun GdbR	RICP-01C0106113	DEU
FORNO	0.78	220	584	0	3	4	40.4	na	*Lr34*	Agroscope/DSP (Federal Research Station for Agronomy)	K-62003; AUS-23641	CHE
ARINA	0.77	220	494	0	1	7	22.4	na	Resistant	Agroscope/DSP (Federal Research Station for Agronomy)	K-57737; K-57528; E-1014; AUS-21732	CHE
AUBUSSON	0.66	217	600	0	5	6	68.7	na	Susceptible	Limagrain Verneuil Hold.	RICP-01C0107106	FRA, ITA
SIMANO	0.76	218	617	1	3	6	24.2	na	Awned	Agroscope/DSP (Federal Research Station for Agronomy)	na	CHE
CH CLARO	0.7	218	556	0	1	5	36.8	na	Check	Agroscope/DSP (Federal Research Station for Agronomy)	na	CHE
ZEBEDEE	0.7	222	624	0	3	9	28.7	na	Other	Nickerson-Advanta Ltd. Advanta Seeds UK Ltd.	AFRC-10073	GBR
OCTET	0.71	220	600	1	1	5	na	na	Other	na	na	FRA

Cultivars were selected to have similar phenology and final height but strongly contrasting canopy architectural and morphological traits. Specifically, the set comprised cultivars with erect and planophile flag leaves and with high and low levels of flag leaf glaucousness.

**GenName**: Cultivar name, **FH**: Final height, **GS55**: Heading date, indicated in days after sowing; **Onsen**: Onset of senescence, indicated in growing degree days after heading; **Awns**: Presence or absence of awns on ears, with ‘0’ indicating absence and ‘1’ indicating presence of awns, **Fl0Ang**: Visual scoring of flag leaf angle, with low values indicating erect flag leaves and high values indicating drooping flag leaves, **Fl0Glc**: Flag leaf glaucousness, with low values indicating low levels of glaucousness and high values indicating high levels of glaucousness, **PLACL**: Percent leaf area covered by lesions, data from Karisto *et al*.^16^, **TraitComb**: Trait combination represented by the cultivar, with ‘L’ and ‘H’ representing low and high values for Fl0Ang and Fl0Glc, respectively, **Criterion**: Selection criterion applied; either the trait combination, the presence of the *Lr34* gene, or the level of resistance. Information on the originator, accession number and cultivar recommendation were taken from the Genetic Resources Information System for Wheat and Triticale (GRIS), at URL http://wheatpedigree.net/, accessed on 28-02-2023. Trait data was based on a field experiment carried out in the wheat growing season of 2018/2019 at the Eschikon site^36^. The table was modified from^2^.

The original main purpose of this dataset was to monitor Septoria tritici blotch lesion expansion with high precision and sufficient throughput to enable quantitative genetics analyses^7,8^. Beyond this, the dataset offers broad opportunities to study the dynamics of disease development under realistic field conditions and to support methodological advances in image-based organ-level stress monitoring.

### Disease dynamics

Using the existing dataset and processing pipeline, additional aspects of epidemic development can be investigated, including lesion, pustule, and fruiting body emergence rates, which can enable an estimation of latent and incubation period duration and their dependence on host genotype and environmental conditions. Many leaves are affected by multiple disorders simultaneously, particularly mixed fungal infections. Accordingly, the dataset provides opportunities for the analysis of dynamic interactions within disease complexes at unprecedented scale and potentially down to the scale of spatially adjacent symptoms^17,18^. These analyses may provide a broader basis for the parameterization and calibration of epidemiological models, enhancing the accuracy and reliability of seasonal epidemic forcasts^19,20^, similar to models developed for single pathosystems^21^. The addition of a temporal dimension is expected to prove useful in confirming or revisiting current interpretations of detailed symptom-level phenotypes, for example those related to the spatial distribution of symptoms on leaves^22^, the density of fruiting bodies within lesions^23^, or the occurrence of chlorotic halos at the lesion perimeter^24^. A single image at a single time point is often insufficient to unambiguously diagnose specific symptoms, since symptom phenotypes can evolve markedly over time^7,25,26^. Image time series offer a powerful tool to better understand the temporal development of visible disease symptoms, which can support the development of early detection and stress diagnosis systems^13,25^.

### Methodological advances

The dataset provides a broad basis for the development of improved methods for leaf image registration, symptom segmentation, and symptom tracking in image time series. Our previous work^7,8^ addressed each of these tasks sequentially (i.e., separately), and solutions to these tasks may also be further improved separately. For example, recently proposed spatially explicit models providing more detailed insights into lesion expansion^27^ could be evaluated and optimized on thousands of lesions located in diverse and complex spatial contexts. Alternatively, recent works in biomedical image processing propose strategies for a joint optimization of several of these steps, highlighting their connectedness. For example, Ren *et al*.^28^ demonstrated that explicitly incorporating spatial and temporal self-similarity between repeated brain scans can improve the performance and longitudinal consistency of self-supervised segmentation models. Similarly, Han *et al*.^29^ showed that jointly learning registration of images from different modalities and image segmentation in a single framework can improve both tasks. Rokuss *et al*.^30^ propose a promptable framework for lesion (tumor) tracking and segmentation in follow-up scans of the same patients. In our context, the existing timepoint-specific segmentation masks could serve as valuable priors to guide such joint optimization approaches for lesion alignment, tracking, and segmentation.

In our previous work, we investigated the predictability of lesion growth based on manually extracted lesion phenotypic traits, experimental design factors, and environmental variables^8^. Other recent work has explored the possibility of modelling the development of disease symptoms directly from image time series^31,32^. Integrating image sequences and environmental data could provide deeper insights into drivers of symptom development, and a corresponding framework has recently been proposed^33^. Our data set provides a solid basis for the evaluation of recent and emerging approaches from other disciplines on image sequences of plant leaves with the aim of achieving more robust and precise tracking of symptom development and a more detailed understanding of drivers of disease progression, based on increasingly less standardized imagery. We believe that precise and robust methods for tracking leaf disorders will not only benefit plant pathologists and epidemiologists but also researchers interested in studying plant responses to other types of stress, such as cold stress, or researchers interested in the spatiotemporal dynamics of developmental processes such as leaf senescence.

## Methods

**Fig. 3:**
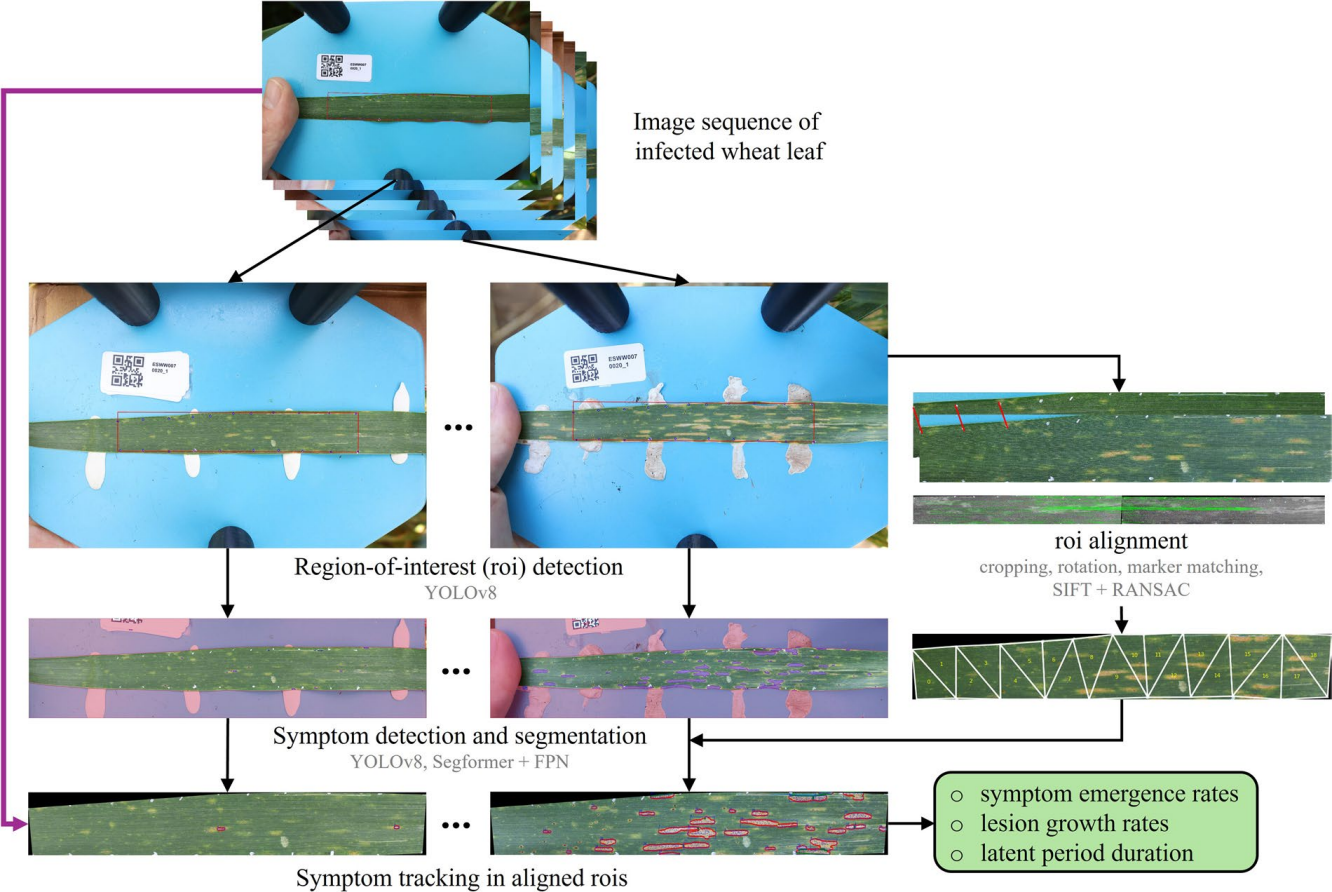
Implemented image processing approach for monitoring of diseases on wheat leaves (black arrows). The region of interest (roi; red rectangle) is identified in each image of a series by detecting manually placed white reference marks. The rois are aligned by matching detected reference marks across frames of an image series to estimate a piece-wise affine transformation. In parallel, original images are processed using key point detection and segmentation models for symptom detection and segmentation. Resulting key point coordinates and segmentation masks are aligned using the parameters estimated in image registration. Symptoms are tracked in a series based on area overlap. This approach delivers useful results but requires manual curation. It overlooks temporal context in image sequences and connections between the tasks of image alignment, image segmentation, and symptom tracking, resulting in suboptimal performance. It could be replaced by a more integrated, potentially end-to-end, framework (purple arrow).

Image sequences were obtained in field experiments during the 2022–2023 and 2023–2024 wheat growing seasons at the ETH Research Station for Plant Sciences at Lindau-Eschikon, Switzerland (47.449°N, 8.682°E, 520 m a.s.l.; soil type: eutric cambisol). Twenty registered European wheat cultivars with contrasting morphology and a broad range of resistance levels were grown in a replicated, factorial design with three main treatments: fungicide plus late inoculation (FI2), early inoculation without fungicide (0FI1), and fungicide without inoculation (F0I). One replicate of an untreated control (0F0I) was also included. Artificial inoculations were made with a mixture of ten *Z. tritici* strains. Air temperature and relative humidity were recorded within the canopy throughout the experiment. Further experimental details are provided elsewhere^8^. All images were collected using a full-frame mirrorless digital camera (EOS R5, Canon Inc., Tokyo, Japan; 45 megapixels, 36- × 24-mm sensor) combined with a macro lens (RF 35 mm f/1.8 IS Macro STM, Canon Inc., Tokyo, Japan). A custom-developed 3D-printed spacer and an acrylic glass base plate as background against which the leaf could be flattened ensured constant working distance and co-planarity of the leaf with the focal plane of the camera^7^. The following camera and lens settings were applied uniformly: manual exposure mode, an exposure time of 1/200 s (increased to 1/160 s if automatic ISO could not be kept below 2000), aperture F7.1, automatic ISO, and an exposure bias of +0.3 or +0.7 step. White balance was set to daylight, autofocus was configured for a large horizontal zone in one-shot mode, and brightness was measured using partial metering. The operator cast a uniform shadow over the field of view. An overview of image processing is given in Fig. 3. Briefly, image registration was based on the detection of artificial reference marks added manually to leaves at the beginning of each measurement series using a white ink marker. Detection was based on a YOLOv8. To train the model, all visible marks in 133 selected example images were annotated as points by clicking once at the estimated centroid of the mark, using the computer vision annotation tool (https://www.cvat.ai). Annotations were exported in ‘CVAT for images 1.1’ format and exported to YOLO-Pose dataset format using a custom-developed python script. The bounding rectangle containing all detected reference marks was then identified and rotated about the center of the image for the bounding box to have an angle of 0°. Matching reference marks across frames of the series were identified and used to estimate piecewise affine transformations. When necessary, alignment was further guided by registering each image to its preceding frame in the series using SIFT feature detection and RANSAC-based feature matching. Further details are provided in earlier work^7^. All images were processed individually with key point detection and segmentation models for symptom detection and segmentation, based on previous work^8,34^. Symptom detection and segmentation was performed on the original images; obtained key point coordinates and segmentation masks were then transformed using the parameters estimated during image registration. Symptoms were tracked in an image series based on area overlap.

## Data Records

The dataset is publicly available from the ETH Zurich publications and research data repository^35^. In addition to the raw images, we provide processed data, including segmentation masks, aligned leaf crops, estimated transformation matrices, and other derived outputs, to facilitate easy reuse of the dataset. However, all processed data can be fully reproduced from the raw images using the accompanying processing scripts, which are publicly available via the associated Git repository.

The dataset is structured first according to the type of data (‘raw’ – all 12,520 RGB images of wheat leaves in.JPG format, ‘processed’ – all processing outputs, and ‘meta’ – meta information). The original full-sized.JPG RGB images in ‘raw’ are organized according to measurement events, which typically correspond to a date of image acquisition (YYYY/YYYYMMDD). Where multiple measurements were made on the same day, measurements are distinguished by an additional ‘_1’ and ‘_2’ suffix. Each RGB image has a unique identifier composed of the timepoint of acquisition (YYYYMMDD_HHMMSS), a plot unique identifier (ESWW00X), and a leaf number. Each of the measurement event folders contains a subfolder ‘runs’ with.txt files that contain the image coordinates of the detected reference marks for each image. The folder ‘processed’ contains all outputs from image alignment and symptom detection, segmentation, and tracking. The two subfolders ‘reg’ and ‘ts’ contain the output from image registration and symptom tracking, respectively. Individual outputs are stored in further subdirectories with self-explanatory names. Both output directories are organized by sample (i.e., by leaf), as identified via the combination of the plot unique identifier (ESWW00X) and the leaf number. This facilitates handling of the data as image sequences. Finally, the folder ‘meta’ contains experimental designs with plot design coordinates, genotype, and treatment information, heading dates, as well as hourly temperature and relative humidity data retrieved from an on-site weather station as.csv files. In addition, incidence and conditional severity data obtained in spring and summer of 2022 and 2023 following a protocol described earlier^1^ are available. Note that there is one year of overlap between reference data and image data, as well as incomplete overlap in terms of investigated genotypes. The subfolder ‘markers’ contains the dataset used to train the reference mark detection model, consisting of images and corresponding marker coordinates in YOLO-pose dataset format.

## Technical Validation

**Fig. 4:**
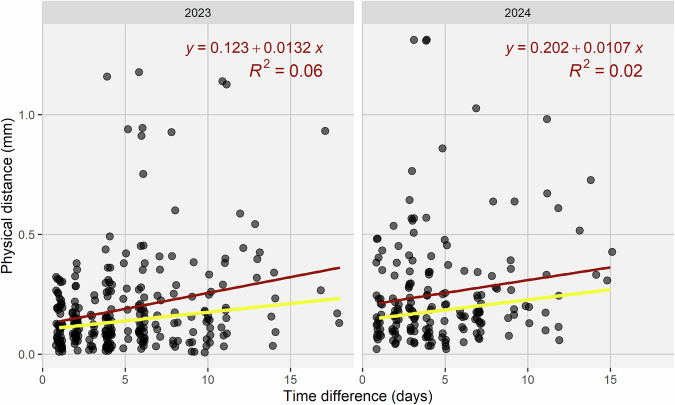
Accuracy of image registration based on manual tracking of prominent features as a function of the temporal lag between pairs of images. The physical distance between objects in randomly sampled pairs of images from image sequences was obtained assuming a pixel size of 0.03 mm. Red lines represent ordinary least squares regression fits; yellow lines represent the median regression (quantile regression at τ = 0.5).

**Fig. 5:**
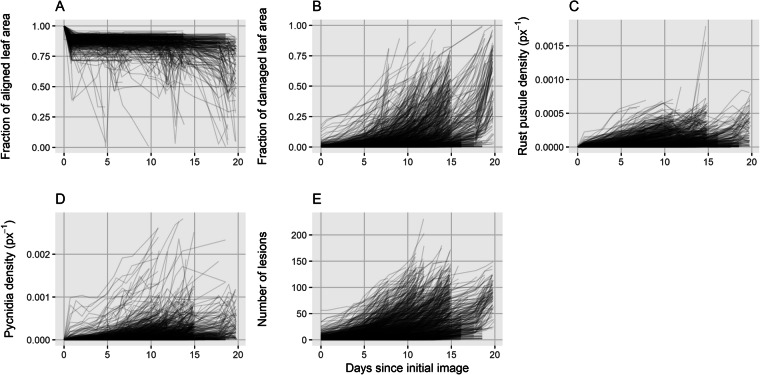
Temporal patterns observed in image sequences. (**A**) Relative leaf area aligned to the initial image of each sequence. (**B**) Damaged leaf area over time for each image sequence. (**C**) Rust pustule density on each leaf over time. (**D**) Pycnidia density on each leaf over time. (**E**) Number of lesions on each leaf.

The quality of image registration was assessed through manual tracking of prominent features in randomly selected image pairs. The median alignment error was 0.16 mm (≈5 pixels; Fig. 4), which is sufficient for reliable lesion tracking^7^. This evaluation ignored occasional image segments that were obviously misaligned and segments for which no alignment was available, for example due to washed-off markers (Fig. 5A). Misaligned segments can be readily identified by comparing the corresponding estimated transformation matrix with those of nearby segments. Pixel-level accuracy was not achieved, and alignment precision may be insufficient to track smaller features such as individual rust pustules or even smaller pycnidia when relying on area overlap, highlighting the interest in improved methods for image alignment and symptom tracking, as discussed above. A large portion of the image sequences was manually reviewed after alignment to help optimize the process of identifying matching pairs of reference marks across consecutive images. Visual review also ensured that sequences were complete, images had been correctly renamed and correctly linked to metadata.

Temporal trends in geometrically aligned leaf area, damaged leaf area, and the counts of necrotic lesions, rust pustules, and pycnidia were examined to identify potential unexpected patterns that might indicate issues with image processing (Fig. 5). Observed trends in leaf area reflect the reduction in analyzable leaf area due to the current image alignment approach, which limits analysis to the region enclosed by the reference marks (initial decrease in leaf area; Fig. 5A). This initial decrease only represents the loss in analyzable leaf area within the bounding box. Significantly larger leaf areas could be analyzed if the need for dense artificial markers would be avoided. Decreases in the density of detected rust pustules (Fig. 5D) mostly indicate issues with rust pustule detection in older leaves.

The accompanying DatasetTools were tested on several randomly selected image series, with the corresponding python script available in the associated Git repository (see next section for details).

## Usage Notes

The dataset comprises leaves from 15 wheat genotypes, which were selected for their different morphology, including leaf visual properties^2^. Inoculations were made with 10 different strains of *Z. tritici*, and substantial natural infection was observed in both growing seasons^8^. Therefore, the dataset captures a broad range of leaf and lesion phenotypes likely to be encountered in similar future studies and methods developed using this dataset are therefore expected to generalize well to new wheat leaf imagery. However, the dataset does not include leaves from other plant species. Applying methods to other crops will therefore likely require additional, comparable datasets, potentially newly created, as no suitable open-source alternatives seem to be currently available. All leaves in the dataset bear artificial reference marks to guide image alignment, image sequences of unmarked leaves are not available. Ideally, improved future methods for image alignment, lesion segmentation, and tracking will eliminate the need for such marks, which are time-consuming to apply and prone to introducing errors, for example when washed off during an imaging campaign. Moreover, their use reduces the analyzable leaf area, since only regions within the marked zone can be reliably aligned^7^.

Significant effort was invested in optimizing parameters related to the detection of matching pairs of reference marks across frames within image sequences to ensure high-quality image alignments^7^. While some alignment errors affecting individual leaf segments remain, these were considered acceptable for our previous applications and are likely acceptable for many future use cases. Nevertheless, manual correction remains possible where residual errors prove problematic. This might be the case when using the dataset to train deep learning frameworks for image alignment, for example.

From a biological and agronomic perspective, the moderate number of cultivars included in the experiment and the single geographic location represent the main limitations of the data set. Though sample sizes are large at the symptom-level and may be further increased with improved methods for image registration and symptom detection, segmentation, and diagnosis, the number of independent environmental scenarios is likely too small to reliably generalize and extrapolate estimated epidemiological parameters. While parameters such as the response of lesion growth to key environmental variables including temperature and relative humidity should be transferrable across environments, the impact of these responses on overall disease development at the cultivar level requires further investigation based on larger data sets involving additional genotypes and environments.

We provide a lightweight Python toolkit to facilitate loading, inspection, and curation of the image sequences and their associated processing products in the associated Git repository (https://github.com/and-jonas/sympathique-wheat).

## Data Availability

The dataset is publicly available from the ETH Zurich publications and research data repository (10.3929/ethz-b-000739194).

## Code availability

Python code for image alignment, and symptom detection, segmentation, and tracking was published previously^7,8,34^. An updated version of the code is open-sourced at https://github.com/and-jonas/sympathique-wheat. The repository contains additional code for loading, inspecting, and editing image series and processing products in the folder ‘DatasetTools’.
